# Remote ischemic conditioning improves myocardial parameters and clinical outcomes during primary percutaneous coronary intervention: a meta-analysis of randomized controlled trials

**DOI:** 10.18632/oncotarget.23818

**Published:** 2017-12-22

**Authors:** Hai Liu, Li Fu, Xiangke Sun, Wei Peng, Zhiwei Chen, Yiliang Li

**Affiliations:** ^1^ Third Department of Cardiac Surgery, First Affiliated Hospital of Zhengzhou University, Zhengzhou 450052, Henan, China; ^2^ Institute of Clinical Medicine, Department of Endocrinology, The Central Hospital of Loudi Affiliated to the University of South China, Loudi 417000, China; ^3^ Department of Cardiology, The Central Hospital of Loudi Affiliated to the University of South China, Loudi 417000, China; ^4^ Department of Neurology, The Third Xiangya Hospital, Central South University, Changsha, Hunan 410013, China; ^5^ Postdoctoral Research Workstation of Neurology, Clinical Medicine, The Third Xiangya Hospital, Central South University, Changsha 410013, China

**Keywords:** remote ischemic conditioning, cardioprotection, clinical outcome, primary percutaneous coronary intervention

## Abstract

We conducted a systematic review and meta-analysis to evaluate the effects of remote ischemic conditioning on myocardial parameters and clinical outcomes in ST segment elevation acute myocardial infarction (STEMI) patients undergoing primary percutaneous coronary intervention. Ten eligible randomized controlled trials with 1006 STEMI patients were identified. Compared with controls, remote ischemic conditioning reduced the myocardial enzyme levels (standardized mean difference =-0.86; 95% CI: -1.44 to -0.28; *P =* 0.004; I^2^ = 94.5%), and increased the incidence of complete ST-segment resolution [odds ratio (OR) = 1.74; 95% CI: 1.09 to 2.77; *P =* 0.02; I^2^ = 47.9%]. Remote ischemic conditioning patients had a lower risk of all-cause mortality (OR = 0.27; 95% CI: 0.12 to 0.62; *P =* 0.002; I^2^ = 0.0%) and lower major adverse cardiovascular and cerebrovascular events rate (OR=0.45; 95% CI: 0.27 to 0.75; *P =* 0.002; I^2^ = 0.0%). Meta-analysis suggested that remote ischemic conditioning conferred cardioprotection by reducing myocardial enzymes and increasing the incidence of complete ST-segment resolution in patients after STEMI. As a result, clinical outcomes were improved in terms of mortality and incidence of major adverse cardiovascular and cerebrovascular events.

## INTRODUCTION

Timely reperfusion to ischemic myocardium after ST segment elevation acute myocardial infarction (STEMI) can result in obvious myocardial ischemia/reperfusion (I/R) injury, which may impair the clinical benefit of primary percutaneous coronary intervention (PPCI) [1]. Considerable effort has been directed at this clinical issue with mixed results, and novel strategies need to be identified.

Remote ischemic conditioning (RIC) was first introduced by Przyklenk et al. in 1993 [2]. By applying several cycles of transient ischemic stimulus in a remote organ (mostly a limb), RIC is known to protect the heart, kidney, and brain from reperfusion injury in various animal models[3]. In humans, RIC was also shown to prevent reperfusion-induced endothelial dysfunction [4, 5], and offers novel, endogenous, noninvasive, and systemic protective potential [6]. In 2010, Bøtker et al. [7] first reported decreased post-PPCI troponin levels and increased myocardial salvage index(MSI) in RIC-treated patients with STEMI, and the use of RIC has been explored in STEMI by other research groups[8, 9]. Recently, several randomized control trials (RCTs) with controversial results have been published [10–12]. We conducted a comprehensive meta-analysis to evaluate the clinical effect of RIC in STEMI patients undergoing PPCI.

## RESULTS

### Study characteristics

**Figure 1:**
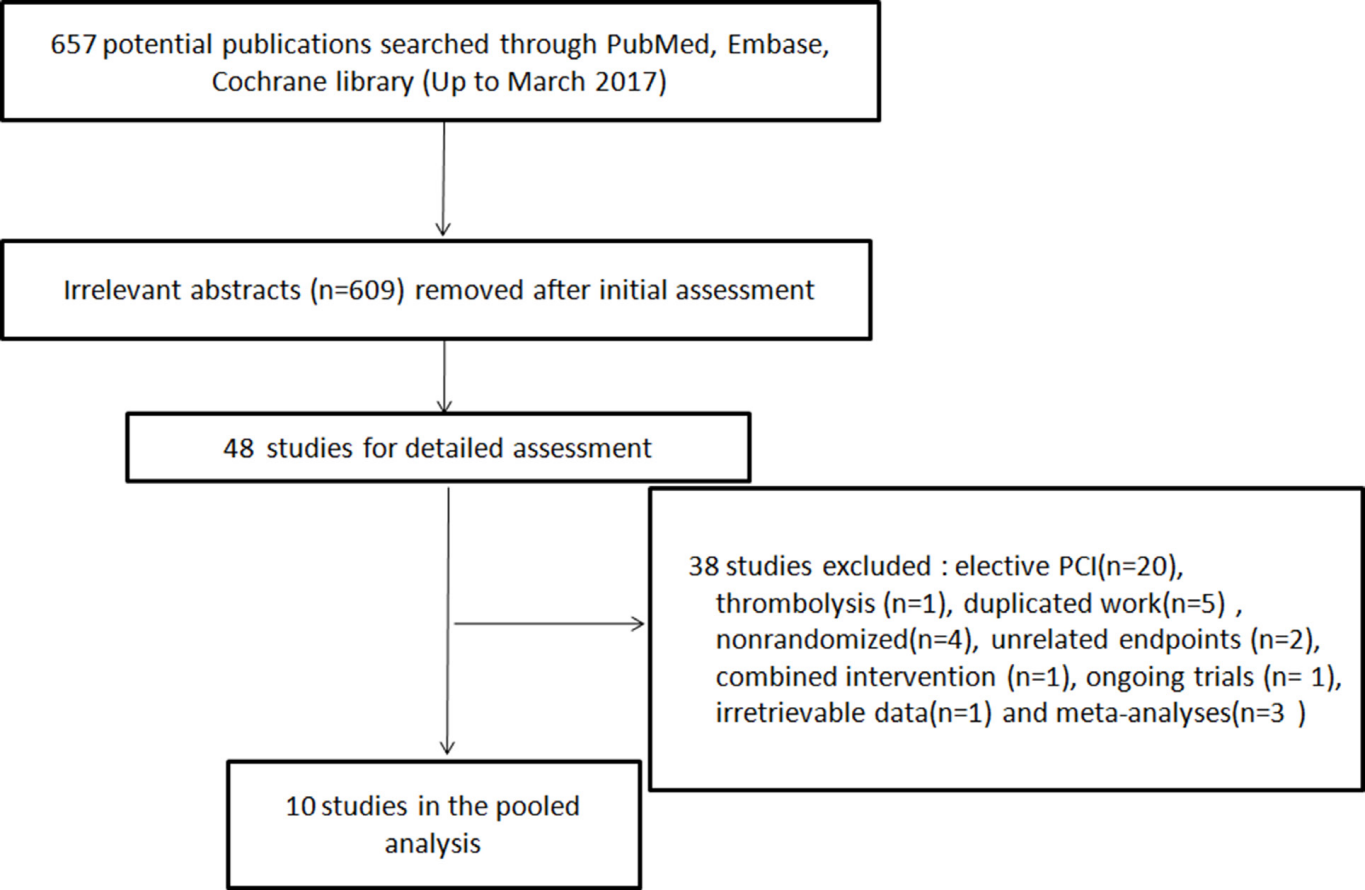
Searching process for eligible studies of remote ischemic conditioning PCI, percutaneous coronary intervention.

Figure 1 shows the process of RCT searching and selection for this study. Ten trials [7–16] enrolling a total of 1006 PPCI patients were included (Figure 1). The ischemic protocol was 3∼4 cycles of 5min/5min in nine studies [7–14, 16] and 3 cycles of 4min/4min in one [15]. The upper limb was used in six studies[7, 8, 11, 12, 15, 16], while the lower limb was used in four studies [9, 10, 13, 14]. For myocardial biomarkers, troponin I or T was used in four studies [7, 8, 10, 15], CK-MB in five studies [9, 11, 13, 14, 16], and CK in one study [12]. The incidence of complete ST-segment resolution (cSTR) was reported in seven trials[7, 9, 11–15]. The MSI was reported in three trials [7, 8, 10]. The imaging infarct size (IS) and myocardial edema were reported in four trials[7–10]. The left ventricular function was reported in six trials [7–12]. For the clinical outcomes, all-cause mortality was reported in five trials[7, 9–12], incidence of heart failure (HF) in three [7, 11, 12], recurrent myocardial infarction (MI) in three [7, 9, 11], target vascular revascularization(TVR) in two [9, 10], stent thrombosis in one [9], stroke in four [7, 9, 11, 12], and major adverse cardiovascular and cerebrovascular events (MACCE) in five [7, 10–12]. The baseline Thrombolysis In Myocardial Infarction (TIMI) flow grade 0∼1 was 100% in four trials[8, 9, 14, 16], partly included in three (58.2% [7], 70.6% [11], 87.1% [10]), and not reported in three [12, 13, 15]. The study characteristics and demographic data are summarized in Supplementary Table 1 and Table 1.

**Table 1:** Patient characteristics in all included randomized trials

Substudy	Age	Male (%)	Pre-MI (%)	DM (%)	HT (%)	Smoking (%)	Dyslip (%)	Multi-vessel (%)	LAD(%)	Direct Stent (%)	β-blockers (%)	Statins (%)	GP(%)
Bøtker 2010 [7]	62.5	75.7	0.0	8.8	31.1	56.2	17.1	31.9	41.0	91.6	15.1	17.3	N.A
Rentoukas 2010 [15]	62.1	60.3	15.9	30.2	46.0	69.8	44.4	N.A	N.A	N.A	98.4	100.0	N.A
Crimi 2013 [9]	58.5	87.5	11.0	11.5	52.0	47.9	31.3	36.5	N.A	N.A	100.0	100.0	94.8
Prunier 2014 [16]	64.0	77.1	0.0	11.4	45.7	34.3	34.3	45.7	40.0	100.0	N.A	N.A	N.A
White 2014 [8]	58.4	80.7	N.A	7.2	24.1	49.4	27.7	N.A	N.A	N.A	N.A	N.A	N.A
Wang 2014 [14]	62.5	76.1	34.8	28.3	65.2	58.7	37.0	84.8	34.8	0.0	100.0	100.0	100.0
Yamanaka 2015 [12]	67.0	76.0	10.6	34.0	63.0	N.A	52.0	N.A	42.0	N.A	5.0	15.5	N.A
Liu 2016 [11]	62.3	79.0	0.0	20.2	42.9	42.9	30.3	67.2	44.5	97.5	N.A	N.A	N.A
Verouhis 2016 [10]	61.0	94.6	0.0	N.A	8.6	22.6	37.6	6.5	94.6	16.1	N.A	N.A	8.6
Gao 2016 [13]	N.A	N.A	N.A	N.A	N.A	N.A	N.A	N.A	N.A	N.A	N.A	N.A	N.A

Note: Pre-MI, previous myocardial infarction; DM, diabetes mellitus; HT, hypertension; Dyslip, dyslipidemia; LAD, left anterior descending; GP, inhibitors of glycoprotein IIb/IIIa; N.A, not available.

### Effect of RIC on postprocedural myocardial parameters

**Figure 2:**
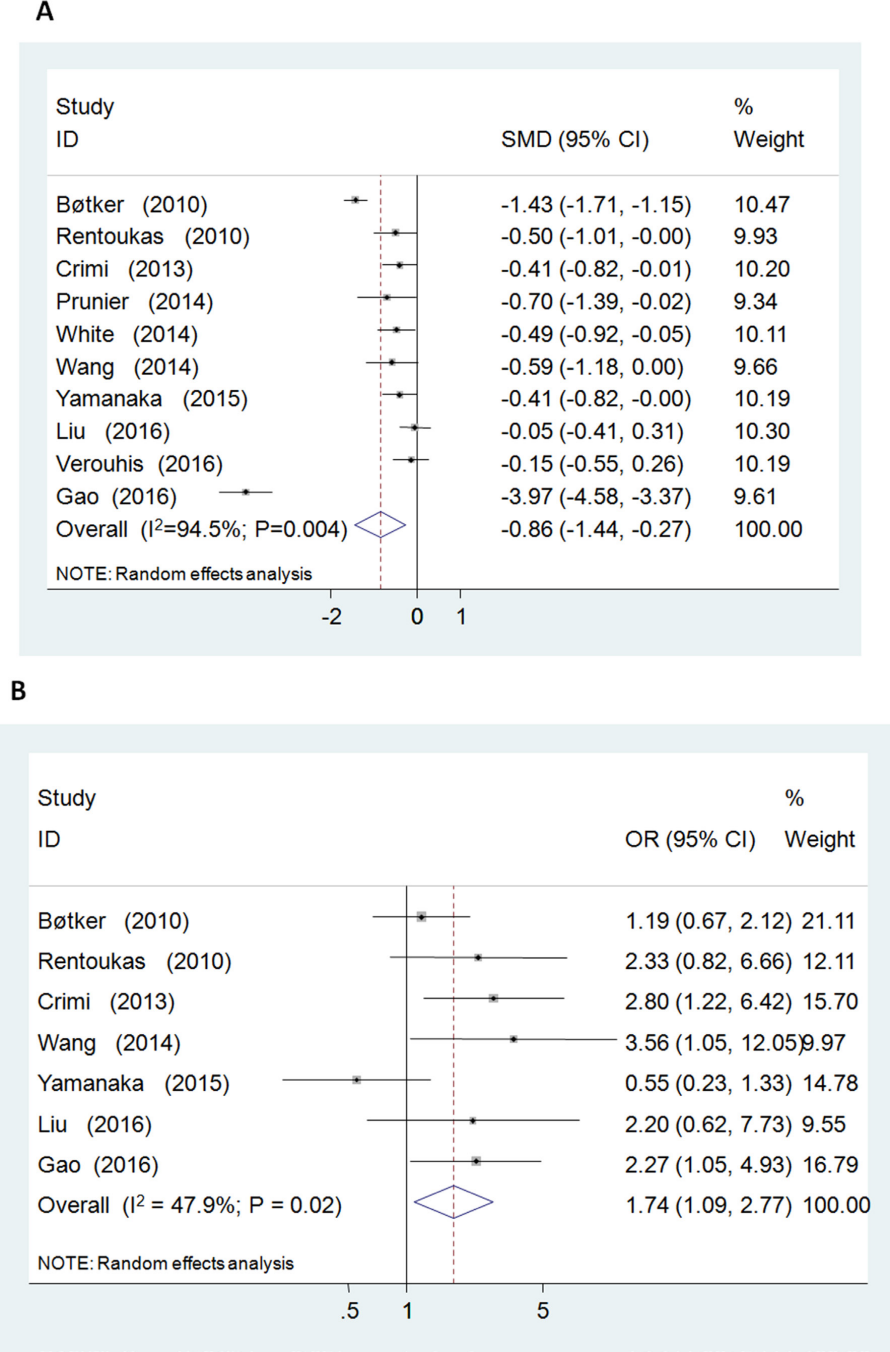
Forest plot for postprocedural myocardial enzyme levels (standardized mean difference = –0.86, P = 0.004; (**A**)) and complete ST-segmental resolution (odds ratio = 1.74, *P* = 0.020; (**B**)).

Postprocedural elevated myocardial enzyme level was reduced by RIC relative to control group [standardized mean differences (SMD) =-0.86; 95% CI: –1.44 to –0.28; *P* = 0.004] with significant heterogeneity (I^2^ = 94.5%) (Figure 2A). No evidence of publication bias was observed (*P* = 0.03, Begg’s test; *P* = 0.75, Egger’s test). Sensitivity analysis excluding each included study one at a time revealed that most individual studies were consistent with the direction and size of the overall myocardial enzymatic effect (All *P* ≤ 0.02) (Figure 4A).

**Figure 3:**
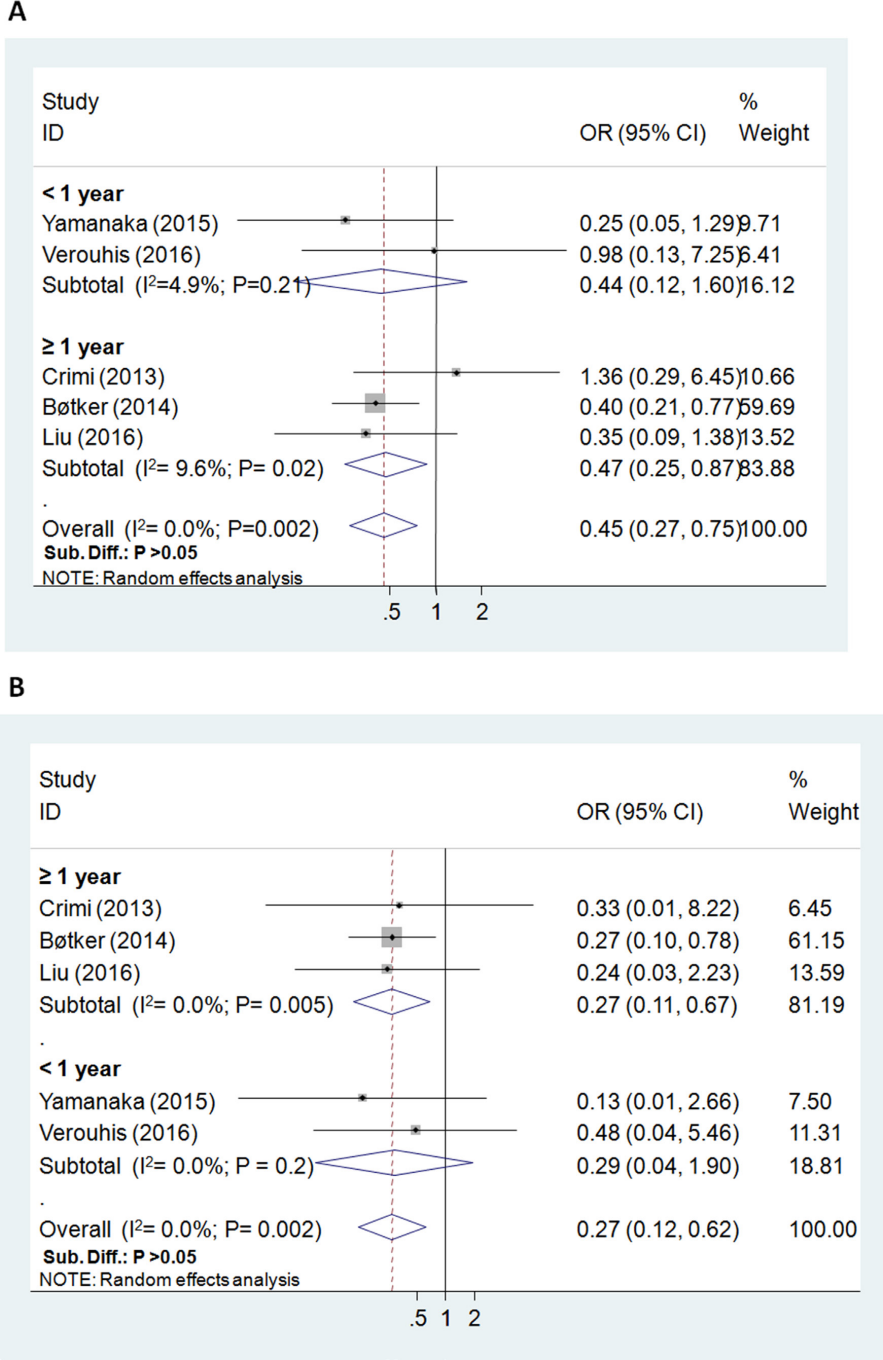
Forest plot for major adverse cardiovascular and cerebrovascular events (odds ratio = 0.45, *P* = 0.002; (**A**)) and all-cause mortality (odds ratio = 0.27, P = 0.002; (**B**)).

**Figure 4:**
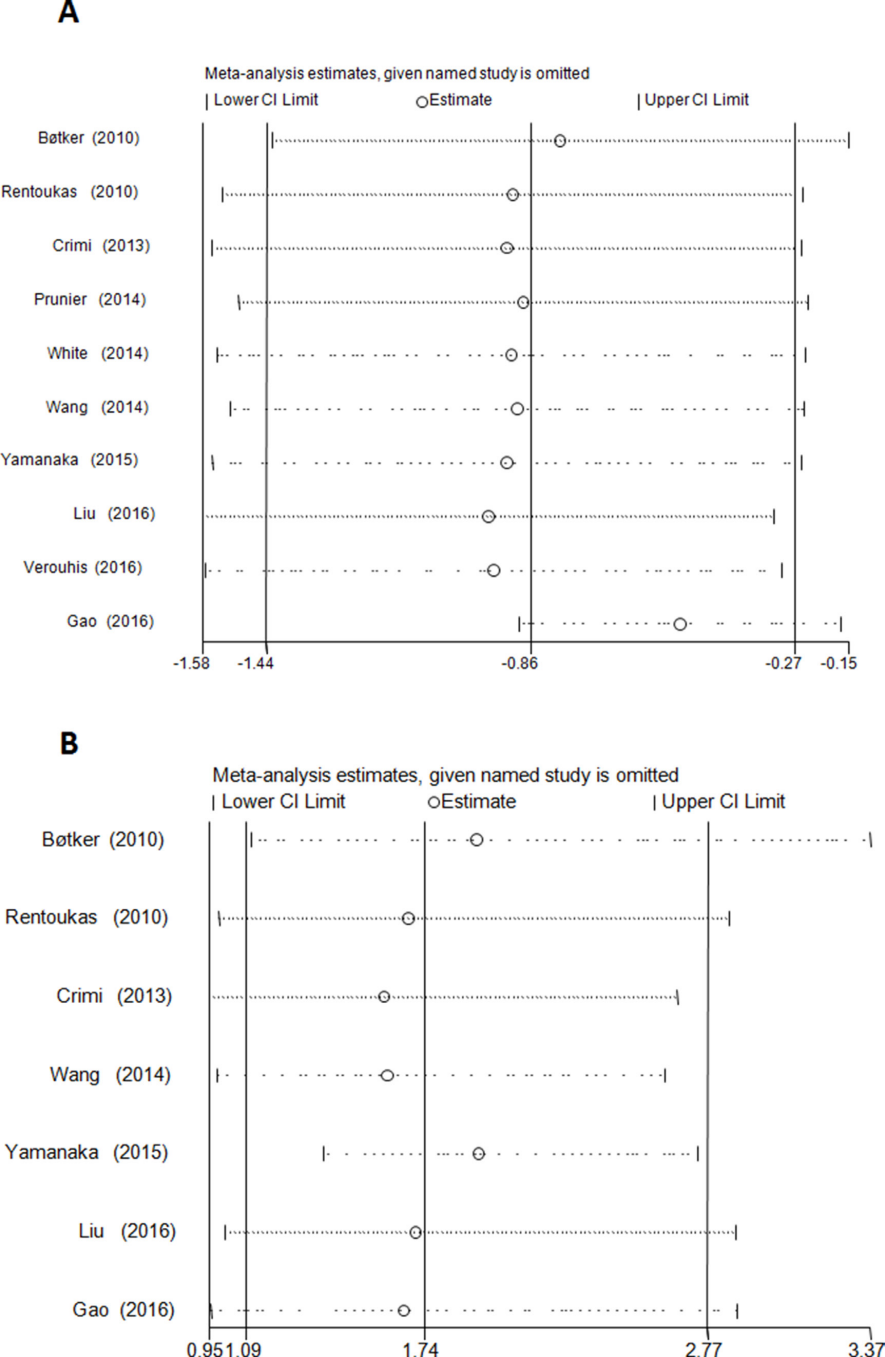
Sensitivity analysis evaluating the influence of each individual study (left side) on the postprocedural myocardial enzyme levels (All *P* ≤ 0.02); (**A**) and complete ST-segmental resolution (All *P* ≤ 0.08); (**B**). The remaining all results with 95% confidence interval (CI) is presented.

RIC increased the post-PPCI rate of cSTR [odds ratio (OR) = 1.74; 95% CI: 1.09 to 2.77; *P* = 0.020; I^2^ = 47.9%] (Figure 2B). No evidence of publication bias was observed (*P* = 1.00, Begg’s test; *P* = 0.35, Egger’s test). Sensitivity analysis excluding each included study one at a time revealed that most individual studies were consistent with the direction and size of the overall cSTR-improving effect (All *P* ≤ 0.08) (Figure 4B).

RIC did not affect the extent of MSI [weighted mean differences (WMD) = 0.09; *P* = 0.16; I^2^ = 93.6], the imaging IS (WMD = -1.62; *P* = 0.29; I^2^ = 73.6%), myocardial edema (WMD = -1.19; *P* = 0.68; I^2^ = 90.6%) or LVEF (WMD = 0.39; *P* = 0.56; I^2^ = 31.4%) (Table 2).

**Table 2:** Pooled analysis of the postoperative myocardial parameters and clinical outcomes

Endpoints	No. of Studies	RIC	Control	OR (95% CI)	SMD/WMD (95% CI)	*P* value
Enzyme level	10	505	501	/	-0.86 (-1.44, -0.28)	0.004
cSTR	7	392	390	1.74 (1.09,2.77)	/	0.020
MSI	3	163	155	/	0.09 (-0.04, 0.23)	0.16
Imaging IS	4	259	268	/	-1.62 (-4.6, 1.36)	0.29
ME	4	193	191	/	-1.19 ( -6.76, 4.39)	0.68
LVEF	6	383	389	/	0.39 (-0.90, 1.67)	0.56
MACCE(overall)	5	323	312	0.45 (0.27, 0.75)	/	0.002
< 1 year	2	94	93	0.44 (0.12, 1.60)	/	0.21
≥1year	3	229	219	0.47 (0.25, 0.87)	/	0.02
All-cause mortality	5	323	312	0.27 (0.12, 0.62)	/	0.002
< 1 year	2	94	93	0.29 (0.04, 1.90)	/	0.20
≥1year	3	229	219	0.27 (0.11, 0.67)	/	0.005
Recurrent MI	4	355	344	0.66 (0.29, 1.51)	/	0.33
TVR	2	95	94	5.72 (0.65, 50.45)	/	0.12
Stent thrombosis	1	48	48	0.32 (0.01, 8.05)	/	0.49
Stroke	4	276	266	0.68 (0.21, 2.21)	/	0.52
HF	3	354	353	0.57 (0.24, 1.34)	/	0.20

Notes: cSTR, complete ST-segmental resolution; MSI, myocardial salvage index; ME, myocardial edema; LVEF, left ventricular ejection fraction; MACCE, major adverse cardiovascular and cerebrovascular event; MI, myocardial infarction; TVR, target vessel revascularization; HF, heart failure; OR, odds ratio; SMD, standardized mean difference; WMD, weighted mean difference; CI, confidence interval; RIC, remote ischemic conditioning.

### Effect of RIC on clinical outcomes

The all-cause mortality (the longest follow-up) was reported in 635 study subjects, and the overall incidence was 5.0% (7/323 in RIC group, 25/312 in control group). Postoperative incidence of death was significantly reduced by RIC (5 RCTs; OR = 0.27; 95% CI: 0.12 to 0.62; *P* = 0.002; I^2^ = 0.0%), , especially in the subgroup with ≥ 1 year follow up (3 RCTs; *P* = 0.005) (Figure 3B).

For MACCE and the composite clinical outcomes, the overall incidence was 12.6% (80/635). The risk of postoperative MACCE was significantly lowered in the RIC group (5 RCTs; OR = 0.45; 95% CI: 0.27 to 0.75; *P* = 0.002; I^2^ = 0.0%), particularly in the subgroup with ≥ 1 year follow up (3 RCTs; *P* = 0.02) (Figure 3A).

The incidence of recurrent MI, TVR, stent thrombosis, stroke, and HF was 4.72% (22/466), 2.65% (5/189), 1.04% (1/96), 1.85% (10/542), and 3.88% (18/464) respectively. There was no significant difference between the RIC group and the control group for these endpoints as shown in Table 2.

## DISCUSSION

In this meta-analysis of ten randomized trials involving 1006 patients, we confirmed that RIC conferred cardioprotection compared to primary PCI alone by reducing elevated myocardial enzyme and increasing the incidence of cSTR in patients after STEMI. No beneficial effect on MSI, myocardial edema, imaging IS, or LVEF was observed. Our study demonstrated that RIC also improved clinical outcomes regarding all-cause mortality and incidence of MACCE.

PCI remains one of the two current cornerstones in the treatment of STEMI patients. However, the incidence of heart failure (≈ 25%) and cardiac death (≈ 10%) after PPCI remains high, partly due to myocardial ischemia/reperfusion (I/R) injury [17]. Paradoxical injury during reperfusion may cause irreversible cardiomyocyte damage and myocardial stunning, resulting in elevated cardiac enzymes, delayed cSTR, and limited myocardial salvage potential [18, 19]. High levels of cardiac enzymes and insufficient cSTR have been recognized as an important predictor of long-term mortality in STEMI patients after PCI [20–22]. Strategies to reduce myocardial enzyme and other myocardial parameters after PCI are critical and may enhance clinical outcomes. The most widely applied type of ischemic conditioning during PPCI is ischemic postconditioning (IPoC) which is performed by intermittent reinflation of the stent balloon immediately after reperfusion (within 1 min). IPoC has been demonstrated to reduce myocardial enzyme levels [23, 24], increase left ventricular function [23], and limit infarct size and myocardial edema [25]. However, the short- and long-term clinical outcomes have been shown to be negative [26–28].

RIC is another endogenous protective approach by inflation of an upper-arm blood pressure cuff, which is more applicable and less harmful than IPoC in clinical settings [6]. Our combined analysis of all negative and positive outcomes showed that RIC exerted cardioprotection regarding enzyme level and cSTR. RIC also reduced all-cause mortality and MACCE risk in STEMI patients. Preventative RIC for acute kidney injury following PPCI was proposed in some clinical studies [12, 29], indicating potential systemic organ protection.

The baseline TIMI flow grade before reperfusion therapy for STEMI has been recognized as an influential factor of post-PPCI myocardial injury [30]. The myocardium of STEMI patients with visualized collateral circulation (TIMI flow grade > 1) in initial coronary angiography are protected and could develop smaller infarct size regardless of protective strategies. It is challenging to evaluate myocardial blood flow in some emergency situations, so these patients should be excluded to explore the protective efficacy of the intervention. The baseline TIMI flow grade was 0∼1 in most of the clinical trials that confirmed the cardioprotection of IPoC in STEMI [23]. Roubille et al. [31] randomized 100 STEMI patients with a TIMI flow grade of 2∼3 and did not identify a cardioprotective effect of IPoC. In our analysis, only four trials excluded the patients with TIMI flow grade > 1, and the cardiac effect was not influenced in other trials that partly included a TIMI flow grade of 2∼3. This phenomenon indicated that RIC might be more useful than IPoC in the clinical setting of PPCI. Future clinical trials should verify the potential advantage of RIC.

In the pooled analysis of the RIC-induced protective effect on myocardial parameters, the value of I-squared for the heterogeneity test ranged from 48.0% to 94.1%. Although the exact sources of significant heterogeneity for ischemic conditioning in this setting remain unknown, previous meta-analysis evidence showed that age (IPoC, STEMI), sex (IPoC, STEMI), stenting technique (IPoC, STEMI) [23], andβ-blockers (RIC, adult cardiac surgery) [32] were influential factors of myocardial protection. However, Bøtker et al. [33–35] used individualized patient data in STEMI and found no impact of cardiovascular factors and medications on the increase in MSI by RIC. Heusch et al.[36] reported similar findings with no evident influential factors for reduction of cardiac troponin I release by RIC in coronary artery bypass graft (CABG). Future work should focus on other potentially influential cardioprotection factors of RIC-treated STEMI such as baseline TIMI flow grade [31], diabetes mellitus [37], and symptom-to-balloon time [38, 39].

The robustness of pooled outcomes in our meta-analysis included the evaluation of publication bias and sensitivity analysis. However, the number of included trials or patients in the analysis may be not adequate to draw a definite conclusion. In our analysis, no evident publication bias was observed in myocardial enzyme level and the incidence of cSTR. Sensitivity analysis confirmed that the contributions of each trial to the overall pooled estimates of myocardial enzyme level and the incidence of cSTR were similar. Future meta-analyses should verify these findings.

Although RIC could elicit cardioprotection in patients with STEMI, the underlying mechanisms are not entirely understood. Various animal and human studies have shown that RIC in the peripheral organs could induce the humoral and neuronal transfer of the protective factors to the remote heart [6, 40]. The reperfusion injury salvage kinase (RISK; PI3K-AKT and ERK1/2) [41], endothelial nitric oxide synthase [42], protein kinase C [43], HIF-1α[44, 45], STAT3 [41], and STAT5 [46, 47] signal pathways in the myocardium have been proposed as contributing mechanisms for the RIC-induced IS reducing effect [48]. Mitochondria are recognized as the end effector of these protective signal pathways during RIC, and the established mechanical sites include permeability transition pores [49], K_ATP_ channels [50], aldehyde dehydrogenase-2 [5], and mitochondrial proteins S-nitrosation [42]. Slagsvold et al. found that RIC improved mitochondrial function in myocardium from the left atrium [51] and left ventricle [52] of patients undergoing on-pump CABG in proof-of-concept clinical studies. These results from animal and clinical studies may explain the effect of RIC in PPCI.

The strengths of this meta-analysis included a comprehensive analysis of clinical outcomes (MACCE), robust approach (sensitivity analysis and publication bias assessment) [53, 54], sufficient clinical consistency (excluding trials concerning thrombolysis [54–56]), and the ability to garner a large study population. There were several limitations in our study. The potential influence of cardiovascular comorbidities and co-medications on the effect of RIC in STEMI patients could not be analyzed from the available individual patient data [23, 32, 57, 58]. The sample size of the included studies was relatively small. The criterion for cSTR was inconsistent among these studies (> 50% in four [9, 11–13], > 70% in two [7, 14], > 80% in one [15]) . The potential confounding effect on the pooled result could not be ruled out. The heterogeneity of enzymatic result was relatively high. Although we used a random-effect model to pool MACCE results, the definitions varied among the included studies. Differences in early and late clinical outcomes require future investigation by large, well-designed, multicenter clinical trials with long-term follow up.

Available evidence from the present meta-analysis suggested that RIC may confer cardioprotection by reducing elevated myocardial enzymes and increasing cSTR incidence in patients after STEMI. Furthermore, our study showed that RIC improved clinical outcomes regarding all-cause mortality and incidence of MACCE.

## MATERIALS AND METHODS

### Search strategy and study criteria

A systematic search was performed according to the PRISMA statement [59] in *PubMed*, *EMBase,* and *Cochrane Library* (up to June 2017) using keyword terms “ischemic conditioning,” “remote ischemic conditioning,” “acute myocardial infarction,” and “percutaneous coronary intervention.” Prospective RCTs enrolling STEMI patients undergoing PPCI were included. The exclusion criteria were: 1) non-English published trials, 2) STEMI patients undergoing thrombolysis, 3) unreported myocardial parameters or clinical outcomes, and 4) RIC combined with other interventions.

### Literature review and data extraction

The literature review and data extraction were independently completed by two investigators (X.S. and W.P.). Discussion was conducted to reach consensus in case of any disagreements. Quality assessment was conducted using Jadad scoring system [60] of randomization, blinding, withdrawals, and dropouts (a possible score between 0 and 5). Trials with a score greater than 3 were considered high-quality.

Data extraction of study characteristics included trial design (year, country, TIMI flow grade, protocol algorithm, follow-up, and symptom-to-balloon time), and patient demographic data (age, sex, diabetes mellitus, hypertension, smoking, dyslipidemia, multi-vessel, left anterior descending artery disease, direct-stenting, β-blockers, statins, and glycoprotein IIb/IIIa inhibitor).

### Postprocedural myocardial parameters

The postprocedural myocardial parameters of interest were as follows: myocardial enzyme levels, the incidence of cSTR, MSI, imaging IS, myocardial edema, and LVEF. The area under the curve (AUC) of total serum troponin or creatine kinase isoenzyme MB (CK-MB) level was preferable in the analysis for its potential superiority. If AUC data were not available, peak troponin or CK-MB or CK level was extracted in turn.

### Clinical outcomes (≥ 1 month follow-up)

The clinical outcomes with ≥ 1 month follow-up were included as follows: all-cause mortality, the incidence of recurrent MI, TVR, stent thrombosis, stroke, and HF. The MACCE were also extracted as a composite endpoint of the included clinical outcomes.

### Data synthesis and analysis

For continuous results (reported with mean and standard deviation or median and interquartile range), we calculated standardized mean differences (SMD) of myocardial enzymes and weighted mean differences (WMD) of LVEF, IS, MSI, and myocardial edema to obtain the pooled estimates with 95% confidence intervals (CI). For dichotomous outcomes (reported with incidence), we calculated the odds ratio (OR) with 95% CI. The random-effect model was used for analysis in consideration of potential clinical inconsistency. Publication bias was assessed by Begg’s and Egger’s test. Sensitivity analysis was used to identify the influence of each included study on the overall estimate of myocardial enzyme and incidence of cSTR. A *P* < 0.05 (2-sided) was considered statistically significant. All statistical analysis was performed with Stata software (version 9.0; Stata Corporation, College Station, TX).

## SUPPLEMENTARY MATERIALS TABLE





## References

[1] Verma S, Fedak PW, Weisel RD, Butany J, Rao V, Maitland A, Li RK, Dhillon B, Yau TM (2002). Fundamentals of reperfusion injury for the clinical cardiologist. Circulation.

[2] Przyklenk K, Bauer B, Ovize M, Kloner RA, Whittaker P (1993). Regional ischemic ‘preconditioning’ protects remote virgin myocardium from subsequent sustained coronary occlusion. Circulation.

[3] Hausenloy DJ, Yellon DM (2008). Remote ischaemic preconditioning: underlying mechanisms and clinical application. Cardiovasc Res.

[4] Loukogeorgakis SP, Panagiotidou AT, Broadhead MW, Donald A, Deanfield JE, MacAllister RJ (2005). Remote ischemic preconditioning provides early and late protection against endothelial ischemia-reperfusion injury in humans: role of the autonomic nervous system. J Am Coll Cardiol.

[5] Contractor H, Stottrup NB, Cunnington C, Manlhiot C, Diesch J, Ormerod JO, Jensen R, Botker HE, Redington A, Schmidt MR, Ashrafian H, Kharbanda RK (2013). Aldehyde dehydrogenase-2 inhibition blocks remote preconditioning in experimental and human models. Basic Res Cardiol.

[6] Heusch G, Botker HE, Przyklenk K, Redington A, Yellon D (2015). Remote ischemic conditioning. J Am Coll Cardiol.

[7] Bøtker HE, Kharbanda R, Schmidt MR, Bottcher M, Kaltoft AK, Terkelsen CJ, Munk K, Andersen NH, Hansen TM, Trautner S, Lassen JF, Christiansen EH, Krusell LR (2010). Remote ischaemic conditioning before hospital admission, as a complement to angioplasty, and effect on myocardial salvage in patients with acute myocardial infarction: a randomised trial. Lancet.

[8] White SK, Frohlich GM, Sado DM, Maestrini V, Fontana M, Treibel TA, Tehrani S, Flett AS, Meier P, Ariti C, Davies JR, Moon JC, Yellon DM (2015). Remote ischemic conditioning reduces myocardial infarct size and edema in patients with ST-segment elevation myocardial infarction. JACC Cardiovasc Interv.

[9] Crimi G, Pica S, Raineri C, Bramucci E, De Ferrari GM, Klersy C, Ferlini M, Marinoni B, Repetto A, Romeo M, Rosti V, Massa M, Raisaro A (2013). Remote ischemic post-conditioning of the lower limb during primary percutaneous coronary intervention safely reduces enzymatic infarct size in anterior myocardial infarction: a randomized controlled trial. JACC Cardiovasc Interv.

[10] Verouhis D, Sörensson P, Gourine A, Henareh L, Persson J, Saleh N, Settergren M, Sundqvist M, Tornvall P, Witt N, Böhm F, Pernow J (2016). Effect of remote ischemic conditioning on infarct size in patients with anterior ST-elevation myocardial infarction. Am Heart J.

[11] Liu Z, Zhao L, Hong D, Gao J (2016). Remote ischaemic preconditioning reduces myocardial ischaemic reperfusion injury in patients with ST-elevation myocardial infarction undergoing primary percutaneous coronary intervention. Acta Cardiol.

[12] Yamanaka T, Kawai Y, Miyoshi T, Mima T, Takagaki K, Tsukuda S, Kazatani Y, Nakamura K, Ito H (2015). Remote ischemic preconditioning reduces contrast-induced acute kidney injury in patients with ST-elevation myocardial infarction: a randomized controlled trial. Int J Cardiol.

[13] Gao J, Liu F, Yang Y, Li X, Zhu J, Ma Y (2016). Remote ischemic per-conditioning of the lower limb during primary percutaneous coronary intervention reduced enzymatice infarct size in STEMI patients. J Am Coll Cardiol.

[14] Wang N, Wang GS, Yu HY, Mi L, Guo LJ, Gao W (2014). [Myocardial protection of remote ischemic postconditioning during primary percutaneous coronary intervention in patients with acute ST-segment elevation myocardial infarction]. [Article in Chinese]. Beijing Da Xue Xue Bao Yi Xue Ban.

[15] Rentoukas I, Giannopoulos G, Kaoukis A, Kossyvakis C, Raisakis K, Driva M, Panagopoulou V, Tsarouchas K, Vavetsi S, Pyrgakis V, Deftereos S (2010). Cardioprotective role of remote ischemic periconditioning in primary percutaneous coronary intervention: enhancement by opioid action. JACC Cardiovasc Interv.

[16] Prunier F, Angoulvant D, Saint Etienne C, Vermes E, Gilard M, Piot C, Roubille F, Elbaz M, Ovize M, Biere L, Jeanneteau J, Delepine S, Benard T (2014). The RIPOST-MI study, assessing remote ischemic perconditioning alone or in combination with local ischemic postconditioning in ST-segment elevation myocardial infarction. Basic Res Cardiol.

[17] Yellon DM, Hausenloy DJ (2007). Myocardial reperfusion injury. N Engl J Med.

[18] Yellon DM, Baxter GF (2000). Protecting the ischaemic and reperfused myocardium in acute myocardial infarction: distant dream or near reality?. Heart.

[19] Ndrepepa G (2015). Improving myocardial injury, infarct size, and myocardial salvage in the era of primary PCI for STEMI. Coron Artery Dis.

[20] Halkin A, Stone GW, Grines CL, Cox DA, Rutherford BD, Esente P, Meils CM, Albertsson P, Farah A, Tcheng JE, Lansky AJ, Mehran R (2006). Prognostic implications of creatine kinase elevation after primary percutaneous coronary intervention for acute myocardial infarction. J Am Coll Cardiol.

[21] Nienhuis MB, Ottervanger JP, Bilo HJ, Dikkeschei BD, Zijlstra F (2008). Prognostic value of troponin after elective percutaneous coronary intervention: A meta-analysis. Catheter Cardiovasc Interv.

[22] de Lemos JA, Braunwald E (2001). ST segment resolution as a tool for assessing the efficacy of reperfusion therapy. J Am Coll Cardiol.

[23] Zhou C, Yao Y, Zheng Z, Gong J, Wang W, Hu S, Li L (2012). Stenting technique, gender, and age are associated with cardioprotection by ischaemic postconditioning in primary coronary intervention: a systematic review of 10 randomized trials. Eur Heart J.

[24] Luo W, Li B, Chen R, Huang R, Lin G (2008). Effect of ischemic postconditioning in adult valve replacement. Eur J Cardiothorac Surg.

[25] Thuny F, Lairez O, Roubille F, Mewton N, Rioufol G, Sportouch C, Sanchez I, Bergerot C, Thibault H, Cung TT, Finet G, Argaud L, Revel D (2012). Post-conditioning reduces infarct size and edema in patients with ST-segment elevation myocardial infarction. J Am Coll Cardiol.

[26] Hahn JY, Yu CW, Park HS, Song YB, Kim EK, Lee HJ, Bae JW, Chung WY, Choi SH, Choi JH, Bae JH, An KJ, Park JS (2015). Long-term effects of ischemic postconditioning on clinical outcomes: 1-year follow-up of the POST randomized trial. Am Heart J.

[27] Hahn JY, Song YB, Kim EK, Yu CW, Bae JW, Chung WY, Choi SH, Choi JH, Bae JH, An KJ, Park JS, Oh JH, Kim SW (2013). Ischemic postconditioning during primary percutaneous coronary intervention: the effects of postconditioning on myocardial reperfusion in patients with ST-segment elevation myocardial infarction (POST) randomized trial. Circulation.

[28] Engstrøm T, Kelbæk H, Helqvist S, Høfsten DE, Kløvgaard L, Clemmensen P, Holmvang L, Jørgensen E, Pedersen F, Saunamaki K, Ravkilde J, Tilsted HH, Villadsen A, Third Danish Study of Optimal Acute Treatment of Patients With ST Elevation Myocardial Infarction–Ischemic Postconditioning (DANAMI-3–iPOST) Investigators (2017). Effect of Ischemic Postconditioning During Primary Percutaneous Coronary Intervention for Patients With ST-Segment Elevation Myocardial Infarction: A Randomized Clinical Trial. JAMA Cardiol.

[29] Crimi G, Ferlini M, Gallo F, Sormani MP, Raineri C, Bramucci E, De Ferrari GM, Pica S, Marinoni B, Repetto A, Raisaro A, Leonardi S, Rubartelli P (2014). Remote ischemic postconditioning as a strategy to reduce acute kidney injury during primary PCI: a post-hoc analysis of a randomized trial. Int J Cardiol.

[30] Ovize M, Baxter GF, Di Lisa F, Ferdinandy P, Garcia-Dorado D, Hausenloy DJ, Heusch G, Vinten-Johansen J, Yellon DM, Schulz R (2010). Postconditioning and protection from reperfusion injury: where do we stand? Position paper from the Working Group of Cellular Biology of the Heart of the European Society of Cardiology. Cardiovasc Res.

[31] Ovize M, Baxter GF, Di Lisa F, Ferdinandy P, Garcia-Dorado D, Hausenloy DJ, Heusch G, Vinten-Johansen J, Yellon DM, Schulz R, Working Group of Cellular Biology of Heart of European Society of Cardiology (2014). No post-conditioning in the human heart with thrombolysis in myocardial infarction flow 2-3 on admission. Eur Heart J.

[32] Zhou C, Liu Y, Yao Y, Zhou S, Fang N, Wang W, Li L (2013). β-blockers and volatile anesthetics may attenuate cardioprotection by remote preconditioning in adult cardiac surgery: a meta-analysis of 15 randomized trials. J Cardiothorac Vasc Anesth.

[33] Pryds K, Terkelsen CJ, Sloth AD, Munk K, Nielsen SS, Schmidt MR, Bøtker HE, CONDI Investigators (2016). Remote ischaemic conditioning and healthcare system delay in patients with ST-segment elevation myocardial infarction. Heart.

[34] Pryds K, Bøttcher M, Sloth AD, Munk K, Rahbek Schmidt M, Bøtker HE, CONDI Investigators (2016). Influence of preinfarction angina and coronary collateral blood flow on the efficacy of remote ischaemic conditioning in patients with ST segment elevation myocardial infarction: post hoc subgroup analysis of a randomised controlled trial. BMJ Open.

[35] Sloth AD, Schmidt MR, Munk K, Schmidt M, Pedersen L, Sørensen HT, Bøtker HE, CONDI Investigators (2015). Impact of cardiovascular risk factors and medication use on the efficacy of remote ischaemic conditioning: post hoc subgroup analysis of a randomised controlled trial. BMJ Open.

[36] Kleinbongard P, Neuhauser M, Thielmann M, Kottenberg E, Peters J, Jakob H, Heusch G (2016). Confounders of Cardioprotection by Remote Ischemic Preconditioning in Patients Undergoing Coronary Artery Bypass Grafting. Cardiology.

[37] Oosterlinck W, Dresselaers T, Geldhof V, Nevelsteen I, Janssens S, Himmelreich U, Herijgers P (2013). Diabetes mellitus and the metabolic syndrome do not abolish, but might reduce, the cardioprotective effect of ischemic postconditioning. J Thorac Cardiovasc Surg.

[38] De Luca G, Suryapranata H, Zijlstra F, van ’t Hof AW, Hoorntje JC, Gosselink AT, Dambrink JH, de Boer MJ, de Boer MJ, ZWOLLE Myocardial Infarction Study Group (2003). Symptom-onset-to-balloon time and mortality in patients with acute myocardial infarction treated by primary angioplasty. J Am Coll Cardiol.

[39] Yetgin T, Magro M, Manintveld OC, Nauta ST, Cheng JM, den Uil CA, Simsek C, Hersbach F, van Domburg RT, Boersma E, Serruys PW, Duncker DJ, van Geuns RJ (2014). Impact of multiple balloon inflations during primary percutaneous coronary intervention on infarct size and long-term clinical outcomes in ST-segment elevation myocardial infarction: real-world postconditioning. Basic Res Cardiol.

[40] Pickard JM, Davidson SM, Hausenloy DJ, Yellon DM (2016). Co-dependence of the neural and humoral pathways in the mechanism of remote ischemic conditioning. Basic Res Cardiol.

[41] Hildebrandt HA, Kreienkamp V, Gent S, Kahlert P, Heusch G, Kleinbongard P (2016). Kinetics and Signal Activation Properties of Circulating Factor(s) From Healthy Volunteers Undergoing Remote Ischemic Pre-Conditioning. JACC Basic Transl Sci.

[42] Rassaf T, Totzeck M, Hendgen-Cotta UB, Shiva S, Heusch G, Kelm M (2014). Circulating nitrite contributes to cardioprotection by remote ischemic preconditioning. Circ Res.

[43] Wolfrum S, Schneider K, Heidbreder M, Nienstedt J, Dominiak P, Dendorfer A (2002). Remote preconditioning protects the heart by activating myocardial PKCepsilon-isoform. Cardiovasc Res.

[44] Cai Z, Luo W, Zhan H, Semenza GL (2013). Hypoxia-inducible factor 1 is required for remote ischemic preconditioning of the heart. Proc Natl Acad Sci U S A.

[45] Albrecht M, Zitta K, Bein B, Wennemuth G, Broch O, Renner J, Schuett T, Lauer F, Maahs D, Hummitzsch L, Cremer J, Zacharowski K, Meybohm P (2013). Remote ischemic preconditioning regulates HIF-1alpha levels, apoptosis and inflammation in heart tissue of cardiosurgical patients: a pilot experimental study. Basic Res Cardiol.

[46] Gedik N, Thielmann M, Kottenberg E, Peters J, Jakob H, Heusch G, Kleinbongard P (2014). No evidence for activated autophagy in left ventricular myocardium at early reperfusion with protection by remote ischemic preconditioning in patients undergoing coronary artery bypass grafting. PLoS One.

[47] Heusch G, Musiolik J, Kottenberg E, Peters J, Jakob H, Thielmann M (2012). STAT5 activation and cardioprotection by remote ischemic preconditioning in humans: short communication. Circ Res.

[48] Skyschally A, Gent S, Amanakis G, Schulte C, Kleinbongard P, Heusch G (2015). Across-Species Transfer of Protection by Remote Ischemic Preconditioning With Species-Specific Myocardial Signal Transduction by Reperfusion Injury Salvage Kinase and Survival Activating Factor Enhancement Pathways. Circ Res.

[49] Turrell HE, Thaitirarot C, Crumbie H, Rodrigo G (2014). Remote ischemic preconditioning of cardiomyocytes inhibits the mitochondrial permeability transition pore independently of reduced calcium-loading or sarcKATP channel activation. Physiol Rep.

[50] Konstantinov IE, Li J, Cheung MM, Shimizu M, Stokoe J, Kharbanda RK, Redington AN (2005). Remote ischemic preconditioning of the recipient reduces myocardial ischemia-reperfusion injury of the denervated donor heart via a Katp channel-dependent mechanism. Transplantation.

[51] Slagsvold KH, Rognmo O, Hoydal M, Wisloff U, Wahba A (2014). Remote ischemic preconditioning preserves mitochondrial function and influences myocardial microRNA expression in atrial myocardium during coronary bypass surgery. Circ Res.

[52] Slagsvold KH, Moreira JB, Rognmo O, Hoydal M, Bye A, Wisloff U, Wahba A (2014). Remote ischemic preconditioning preserves mitochondrial function and activates pro-survival protein kinase Akt in the left ventricle during cardiac surgery: a randomized trial. Int J Cardiol.

[53] Gao J, Chen Q, Liu F, Zhao Q, Chen B, Zhou Y, Ma Y, Yang Y (2017). The effects of remote ischemic conditioning in patients with ST-segment elevation myocardial infarction treated with primary percutaneous coronary intervention: a meta-analysis. Minerva Med.

[54] Man C, Gong D, Zhou Y, Fan Y (2017). Meta-analysis of remote ischemic conditioning in patients with acute myocardial infarction. Sci Rep.

[55] Yellon DM, Ackbarkhan AK, Balgobin V, Bulluck H, Deelchand A, Dhuny MR, Domah N, Gaoneadry D, Jagessur RK, Joonas N, Kowlessur S, Lutchoo J, Nicholas JM (2015). Remote Ischemic Conditioning Reduces Myocardial Infarct Size in STEMI Patients Treated by Thrombolysis. J Am Coll Cardiol.

[56] Ghaffari S, Pourafkari L, Manzouri S, Nader ND Effect of remote ischemic postconditioning during thrombolysis in STEMI. Herz.

[57] Zhou C, Li L (2012). Age may contribute to the negative cardiac effect of postconditioning on STEMI patients. Int J Cardiol.

[58] Ferdinandy P, Hausenloy DJ, Heusch G, Baxter GF, Schulz R (2014). Interaction of risk factors, comorbidities, and comedications with ischemia/reperfusion injury and cardioprotection by preconditioning, postconditioning, and remote conditioning. Pharmacol Rev.

[59] Moher D, Liberati A, Tetzlaff J, Altman DG, PRISMA Group (2009). Preferred reporting items for systematic reviews and meta-analyses: the PRISMA statement. PLoS Med.

[60] Jadad AR, Moore RA, Carroll D, Jenkinson C, Reynolds DJ, Gavaghan DJ, McQuay HJ (1996). Assessing the quality of reports of randomized clinical trials: is blinding necessary?. Control Clin Trials.

