# Supplementary material for: Remote ischemic conditioning improves myocardial parameters and clinical outcomes during primary percutaneous coronary intervention: a meta-analysis of randomized controlled trials
Source: Oncotarget. 2017 Dec 22;9(9):8653–64. doi: 10.18632/oncotarget.23818 (PMC5823569; doi:10.18632/oncotarget.23818)
Supplement: Supplementary file 1 [file oncotarget-09-8653-s001.pdf]

Remote ischemic conditioning improves myocardial parameters and clinical outcomes during primary percutaneous coronary intervention : A meta-analysis of randomized controlled trials

SUPPLEMENTARY MATERIALS

Supplementary Table 1: Study design in all included randomized trials. See Supplementary_Table 1
