# Supplementary material for: Remote ischemic conditioning improves myocardial parameters and clinical outcomes during primary percutaneous coronary intervention: a meta-analysis of randomized controlled trials
Source: Oncotarget. 2017 Dec 22;9(9):8653–64. doi: 10.18632/oncotarget.23818 (PMC5823569; doi:10.18632/oncotarget.23818)
Supplement: Supplementary file 2 [file oncotarget-09-8653-s002.doc]

**Supplementary Table 1: Study design in all included randomized trials.**

| **Study** | **Country** | **PCI type** | **TIMI flow 0~1** | **Symptom- to-balloon time (h)** | **Pts. No.**  **RIC vs Ctrl** | **RIC protocol** | | **RICfirst inflation** | **Placebo**  **Control** | **Endpoints** | **Follow-up** | **MACCE Definition** | **Jadad score** |
| --- | --- | --- | --- | --- | --- | --- | --- | --- | --- | --- | --- | --- | --- |
|  |  |  |  |  |  | **Cycles×I/R Cuff pressure** | |  |  |  |  |  |  |
| Bøtker 2010[7] | Denmark | Primary PCI | 58.17% | 3.15 | 126 vs 125 | 4×5min/5min at upper limb | 200mmHg | >40 min before hospital | No | cTnT, LVEF, IS, ME,MSI, cSTR, MACCE | 3.8 years | All-cause Mortality, MI, HF, Stroke | 3 |
| Rentoukas 2010[15] | Greece | Primary PCI | N.A | 3.21 | 33 vs 30 | 3×4min/4min at upper limb | SBP+20mmHg | 10 min before first balloon | Yes | cTnI, cSTR | Inhospital | / | 1 |
| Crimi 2013[9] | Italy | Primary PCI | 100% | 3.01 | 48 vs 48 | 3×5min/5min at lower limb | 200mmHg | At first balloon | No | CKMB, LVEF, IS, ME, cSTR, MACCE | 1 year | All-cause Mortality, MI, HF, Stroke, TVR | 3 |
| Prunier 2014[16] | France | Primary PCI | 100% | 3.9 | 18 vs 17 | 3×5min/5min at upper limb | 200mmHg | 30min before first balloon | No | CKMB | Inhospital | / | 3 |
| White 2014[8] | UK | Primary PCI | 100% | 3.1 | 43 vs 40 | 3×5min/5min at upper limb | 200mmHg or SBP+ 15mmHg | Before first balloon | Yes | cTnT, LVEF, IS, ME,MSI | Inhospital | / | 5 |
| Wang 2014[14] | China | Primary PCI | 100% | 4.3 | 23 vs 23 | 3×5min/5min at upper limb | 200mmHg | After first balloon(<1min) | No | CKMB, cSTR | Inhospital | / | 2 |
| Yamanaka 2015[12] | Japan | Primary PCI | N.A | 5.72 | 47 vs 47 | 3×5min/5min at upper limb | 200mmHg | 30min before first balloon | Yes | CK, LVEF, cSTR, MACCE | 1 month | Unstable angina  or acute coronary syndrome, heart failure, cardiac rupture, cardiac death, stroke, and transient  ischemic attack | 5 |
| Liu 2016[11] | China | Primary PCI | 70.6% | 6.85 | 60 vs 59 | 4×5min/5min at upper limb | 200mmHg | >40 min before hospital | No | CKMB, LVEF, cSTR, MACCE | 1 year | All-cause mortality, MI, Heart failure, stroke, and transient ischemic attack | 3 |
| Verouhis 2016[10] | Sweden | Primary PCI | 87.1% | 2.42 | 47 vs 46 | 5~6×5min/5min at thigh | 200mmHg or SBP+ 20mmHg | >10min before first balloon | Yes | cTnT, LVEF, IS, ME,MSI, MACCE | 1 month | All-cause mortality, TVR | 5 |
| Gao 2016[13] | China | Primary PCI | N.A | N.A | 60 vs 66 | 3×5min/5min at lower limb | 200mmHg | Before first balloon | N.A | CKMB, cSTR, | Inhospital | / | N.A |

Note:PCI, percutaneous coronary intervention; TIMI, Thrombolysis In Myocardial Infarction; I/R, ischemia/reperfusion; SBP, systolic blood pressure; LVEF, left ventricular ejection fraction; IS, infarct size; ME, myocardial edema; MSI, myocardial salvage index; cSTR, complete ST-segmental resolution; MACCE, major adverse cardiovascular and cerebrovascular events; MI, myocardial infarction; HF, heart failure; TVR, target vessel revascularization; N.A, not available; RIC, remote ischemic conditioning; Ctrl, control.
